# Notch Effects on the Stress Intensity Factor and on the Fatigue Crack Path for Eccentric Circular Internal Cracks in Elliptically Notched Round Bars under Tensile Loading

**DOI:** 10.3390/ma15249091

**Published:** 2022-12-19

**Authors:** Jesús Toribio, Beatriz González, Juan-Carlos Matos, Iván González

**Affiliations:** Fracture & Structural Integrity Research Group (FSIRG), University of Salamanca (USAL), E.P.S., Campus Viriato, Avda. Requejo 33, 49022 Zamora, Spain

**Keywords:** notch effect, elliptical notched bar, eccentric circular inner crack, finite element method, *J*-integral, stress intensity factor (SIF), fatigue propagation, fatigue crack path, constraint loss

## Abstract

In this paper, stress intensity factor (SIF) solutions are numerically obtained for notched bars subjected to tensile loading containing an eccentric circular inner crack located in the cross-section corresponding to the notch root. The finite element method and the *J*-integral have been used to obtain the SIF and to analyze the effect on it of three elliptical notch geometries (of equal radial depth). The results show how the SIF is greater in the notched bars than in the smooth bar and within the former when the axial semi-axis of the notch rises, its effect being greater as the diameter and eccentricity of the inner crack increase. In addition, the fatigue growth of an eccentric crack induces an increase in such eccentricity, greater as the notch axial semi-axis increases. The cause of these phenomena can be attributed to the constraint loss caused by the notch, which also facilitates bending of the specimen due to the asymmetry generated by the crack eccentricity.

## 1. Introduction

Internal cracks can appear in materials as defects during the manufacturing process [1] or caused by phenomena such as high or very high cycle fatigue due to internal inclusions, producing optically dark areas (ODA) and fish-eye patterns [2,3]. In additive manufacturing (AM) technology, considered one of the most promising manufacturing technologies, components are known to exhibit various internal defects such as powder agglomeration, balling, porosity, internal cracks, etc. [4]. In addition, for shot-peened specimens, fatigue fracture did not take place from the surface due to high compressive residual stress on the surface [5]. The carburizing heat treatment also causes the fatigue failure mode of steel changes from surface failure to internal failure [6].

In this framework, the stress intensity factor (SIF) is of great interest. SIF solutions applicable to circular inner cracks located inside a bar subjected to tensile loading have been obtained in the past for the symmetrical case [7,8,9,10,11] and when the circular cracks exhibit eccentricity in relation to the bar axis, it is for the non-symmetrical case [12,13,14,15]. The finite element method (FEM), and others derived from it (extended finite element method, XFEM; scaled boundary finite element method, SBFEM; combined finite-discrete element method, FEM-DEM; combined damage mechanics method and XFEM, CDM-XFEM, etc.), have been used to numerically study the fracture behavior of cracked solids, simulate crack propagation, and study impact fracture and fragmentation processes [16,17,18]. These methods introduce certain advantages over FEM, related in many cases to the decrease in implementation difficulty and computational cost.

In a crack growing from an initial defect, a quick tendency towards a crack front of circular or elliptical geometry is observed. A numerical study of the fatigue propagation of an inner crack with the geometry of a small circle in a round bar showed that it propagates retaining its original shape, but as it approaches the free surface, the part of the adjacent front grows faster, finally leading to a slight distortion of such circular geometry [19]. In round bars that contain circular inner cracks and are subjected to tensile stress, an increase of relative crack eccentricity and of relative crack diameter raises the difference between the SIF values (caused by eccentricity) at the crack point closest to the bar surface and the crack point furthest from it [14].

With the study of the SIF for elliptical surface cracks in notched round bars, it was concluded that the SIFs are strongly influenced by the stress concentration coefficient (especially near the notch root) and nearly independent of the notch geometry (for a given stress concentration coefficient) [20]. The notch effect on the SIF values and on the fatigue behavior is significant in round bars under mode I loading with an elliptical-arc surface crack at the notch root [21].

The notch structural stress method (verified by double V-notch fatigue testing) reflects the notch effect on fatigue crack propagation life, which is based on a closed-form stress solution along the ligament and an analytical *K*_I_ solution for the crack propagating under the notch effect [22]. In Ni-superalloy bars subjected to high cycle fatigue, it has been observed that multiple primary cracks initiate at the surface in notched bars and at the carbide/matrix interface in smooth bars [23]. However, the experimental study of the fracture and propagation of internal cracks is sometimes complicated due to the difficulty of their manufacture in a concrete position of the solid. These cracks have been carried out in transparent solids using laser technology [24].

The simulation of the crack propagation path according to the Paris law [25] requires knowing the SIF value or the energy release rate value along the crack front. The energy release rate, computed using the VCCT 3D FEM-based virtual crack-closure technique, together with the Paris law, showed acceptable agreement compared to experimental fatigue investigation of S355 and S960 structural steel [26]. In addition, considering the Paris–Hertzberg law and knowing the SIF in some points of the crack front, the growth of fish-eye cracks in very high cycle fatigue has also been modelled [27]. In bars subjected to cyclic tensile loading, the existence of eccentricity in the initial circular inner crack leads to an increase of this parameter when fatigue propagation occurs [15].

The aim of this work is to study how the existence of a notch influences the SIF values and the fatigue crack paths of circular internal cracks contained in the cross-section of notched bars under axial tensile loading, when the cracks have a certain eccentricity in relation to the wire axis. The analysis has been performed for three elliptical notch geometries (of equal depth and different ratio between semi-axes) and the results are compared with those of the smooth bar. The relevance of this research lies in the fact that there are always internal defects in materials susceptible to produce, by different mechanisms, an internal crack or behave as such, and its novelty is that there are hardly any scientific studies on the effect produced by a notch on its fracture behavior.

## 2. Numerical Procedure

Using the finite element method (FEM) with the commercial program MSC.Marc, the SIF *K*_I_ along the crack front was computed for eccentric circular inner cracks (i.e., penny-shape cracks) placed in the cross-section corresponding to the root of an elliptical notch in a bar subjected to axial tensile loading. The results were compared with those associated to the cracked smooth bar (without notch) with the same external diameter.

**Figure 1:**
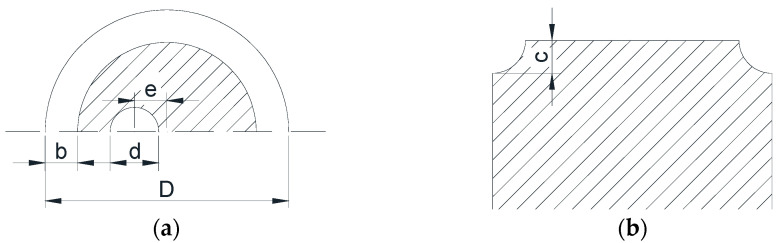
Parameters defining the geometry of the elliptical notched bar with an eccentric circular inner crack: (**a**) cross-section containing the crack; (**b**) longitudinal section of the bar.

In the notched bar, of 3 mm of external diameter (*D*), three elliptical notch geometries were considered, with the same semi-axis *b* (radial direction) of 0.4 mm and different semi-axis *c* (axial direction). They were denominated: sharp notch *c*/*b* = 0.5, circular notch *c*/*b* = 1, and blunt notch *c*/*b* = 2. The geometry of the circular inner crack was characterized by its diameter *d* and eccentricity *e* with respect to the bar axis (Figure 1), the values analyzed being those corresponding to Table 1. In the cracked smooth bar, the same cases were studied as for the cracked notched bars.

**Figure 2:**
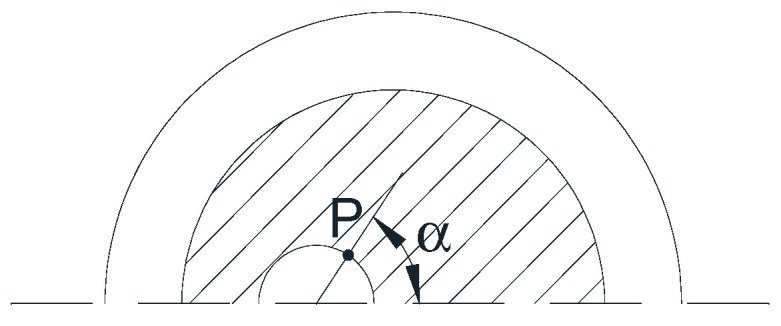
Angle *α* defining a point *P* of the crack front.

Each point *P* of the crack front has been marked by the angle *α* corresponding to the arc of the circle between the point of the crack front farthest from the free surface and the point *P* itself (see scheme in Figure 2).

**Figure 3:**
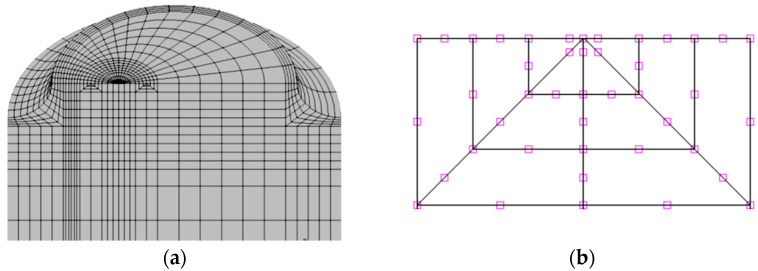
Finite element mesh: (**a**) 3D general view; (**b**) crack tip detail.

In the finite element analysis, and due to the problem symmetry, the mesh has been modeled representing a quarter of the bar with the appropriate boundary conditions; this was further refined in the areas near the crack front and the notch (Figure 3a). Twenty-node isoparametric hexahedron elements were used and the nodes closest to the crack front were shifted to the ¼ position to represent the stress singularity (Figure 3b).

The SIF *K*_I_ was obtained from the energy release rate *G* [28], evaluated through the *J*-integral, considering plane strain conditions and the mechanical properties characteristic of a steel (Young’s modulus *E* = 200 GPa and Poisson’s ratio *ν* = 0.3):

(1) $$G=\frac{K_{I}^{2}}{E/\left(1-\nu^{2}\right)}$$

The dimensionless SIF *Y* was calculated with the following expression, where *σ* is the remote tension stress applied to the ends of the bar:

(2) $$Y=\frac{K_{I}}{\sigma \sqrt{\pi D}}$$

**Figure 4:**
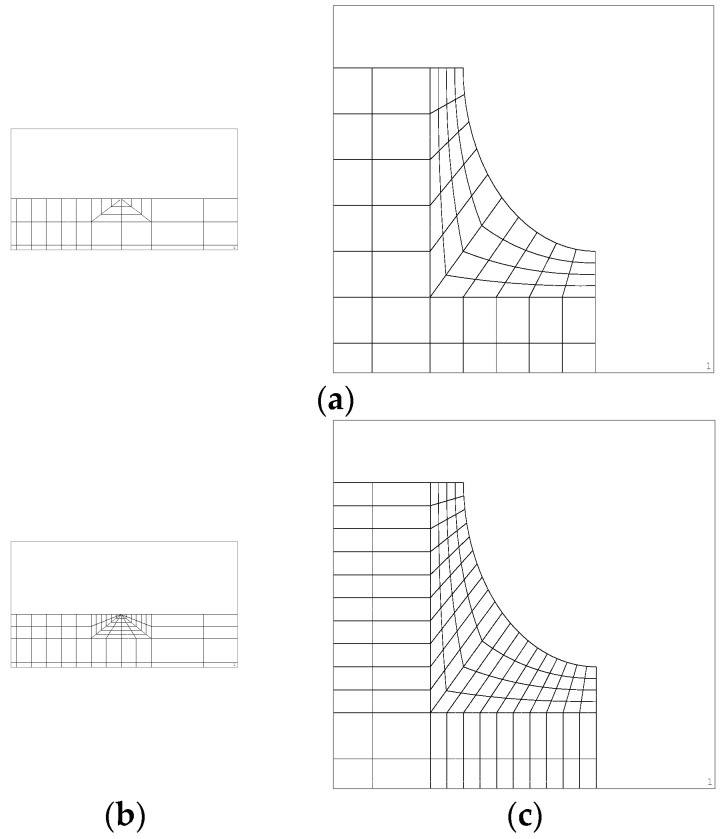
Mesh details: (**a**) crack tip and notch area of the mesh A; (**b**) mesh B, more refined at the crack tip; (**c**) mesh C, more refined around the notch.

A convergence study of mesh size was performed. Bars with a circular notch containing internal cracks with relative crack diameter *d*/*D* = 3/30 and 13/30 and presenting the maximum eccentricity studied *e*/*D* = 6/30 and 1/30, respectively, have been used in the calculations. To this end, in addition to the meshes corresponding to the results presented in this paper (mesh A), meshes were made with further refinement at the crack end (mesh B) and more refined ones around the notch (mesh C). Figure 4 shows some details of these meshes and Table 2 indicates the number of elements for each of them.

**Figure 5:**
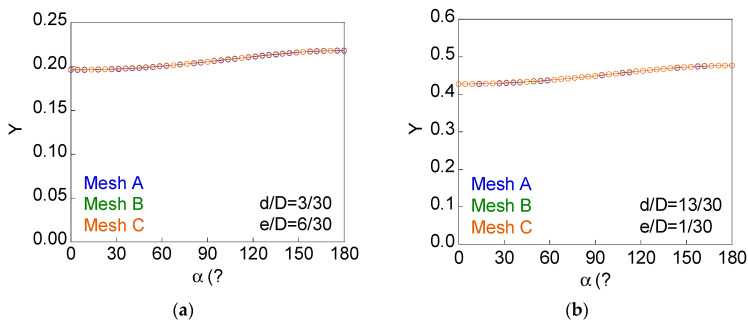
Dimensionless SIF along the crack front for *c*/*b* = 1 (convergence study of the mesh size): (**a**) *d*/*D* = 3/30 and *e*/*D* = 6/30; (**b**) *d*/*D* = 13/30 and *e*/*D* = 1/30.

Figure 5 represents the dimensionless SIF along the crack front (*Y*-*α* curve) for a circular notch and circular inner crack geometries with *d*/*D* = 3/30 − *e*/*D* = 6/30 and *d*/*D* = 13/30 − *e*/*D* = 1/30. Meshes A, B, and C were used for the calculations, with a very good agreement in the results.

## 3. Numerical Results and Discussion

### 3.1. Stress Intensity Factors for the Symmetrical Case

**Figure 6:**
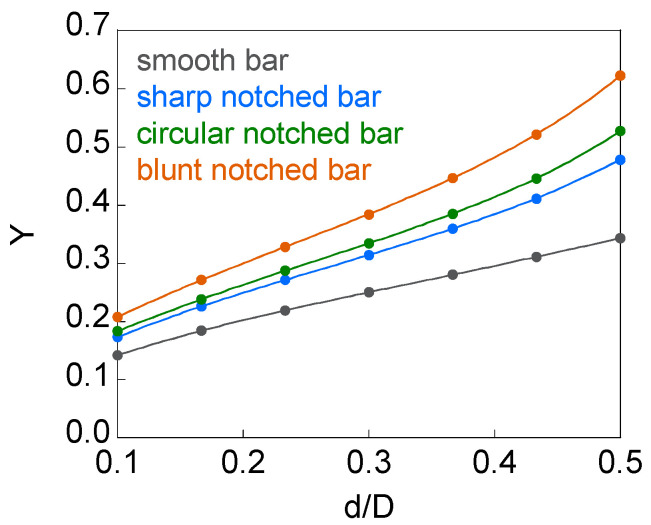
Dimensionless SIF vs. relative crack diameter for smooth and notched bars with non-eccentric circular inner cracks (symmetrical case).

Figure 6 shows the variation of the dimensionless SIF *Y* with the relative crack diameter *d*/*D* (varying from 0.1 to 0.5) in non-eccentric circular inner cracks located in the cross-section of a smooth bar and of elliptically notched bars (with *c*/*b* = {0.5, 1, and 2}). These geometries present symmetry of revolution and correspond to the symmetric case.

The result for the symmetric cracked smooth bar case fits well with the solution provided in the handbook of Tada, Paris, and Irwin [8] and in the handbook of Murakami [9], obtained with the asymptotic approximation method:

(3) $$K_{I}=K_{0}\frac{{\left(1-\left(d/D\right)\right)}^{1/2}}{1-{\left(d/D\right)}^{2}}\left[1+\left(1/2\right)\left(d/D\right)-\left(5/8\right){\left(d/D\right)}^{2}+0.421{\left(d/D\right)}^{3}\right]$$

where *K*_0_ (reference value) is the SIF when the crack is in an infinite medium:

(4) $$K_{0}=\frac{2}{\pi }\sigma \sqrt{\pi \frac{d}{2}}$$

The dimensionless SIF *Y* increases with the relative crack diameter *d*/*D* (Figure 6), its value being greater for the notched specimens than for the smooth ones and, within the former, it increases with the notch axial semi-axis *c*. Therefore, the SIF decreases with the stress concentrator factor *K*_t_ contrary to what happens in the outer circumferential cracks [29]. The effect of the notch on the SIF rises as the crack front approaches the free surface with increasing crack diameter *d*/*D*. The SIF results are closer between the sharp notched bar (*c*/*b* = 0.5) and the circular notched bar (*c*/*b* = 1) than between the circular notched bar (*c*/*b* = 1) and the blunt notched bar (*c*/*b* = 2), and these latter ones are closer than the SIFs between the smooth bar and sharp notched bar (*c*/*b* = 0.5). The effect of the notch on the SIF can be associated with the constraint loss on the crack front produced by the notch, the constraint becoming smaller as the axial semi-axis *c* of the notch increases (greater lack of material in the specimen in relation to the smooth bar).

In round bars, the notch effect on the SIF for the circular inner crack differs quite a bit from that for the outer circumferential (annular) crack. For circular inner cracks, the SIF is higher for notched bars than for smooth bars (even for small relative crack diameters), the effect of the notch on the SIF being greater as the relative crack diameter increases, i.e., as the crack front approaches the notch. For outer circumferential cracks, the SIF is lower in notched bars than in the smooth bar (the crack depth is smaller in the notched bars than in the smooth bar when the outer diameter is the same) and, as the crack depth increases, the SIF values converge to those of the cracked smooth bar [29].

### 3.2. Stress Intensity Factors for Eccentric Circular Inner Cracks

**Figure 7:**
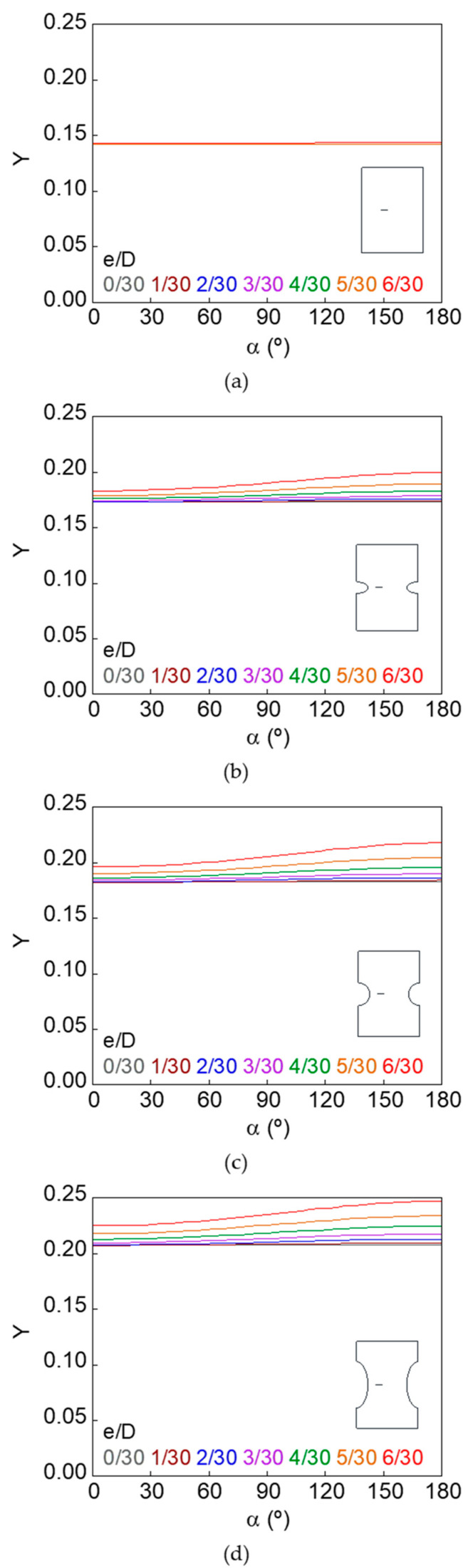
Dimensionless SIF along the crack front, *d*/*D* = 3/30: (**a**) smooth bar; (**b**) sharp notched bar; (**c**) circular notched bar; (**d**) blunt notched bar.

**Figure 8:**
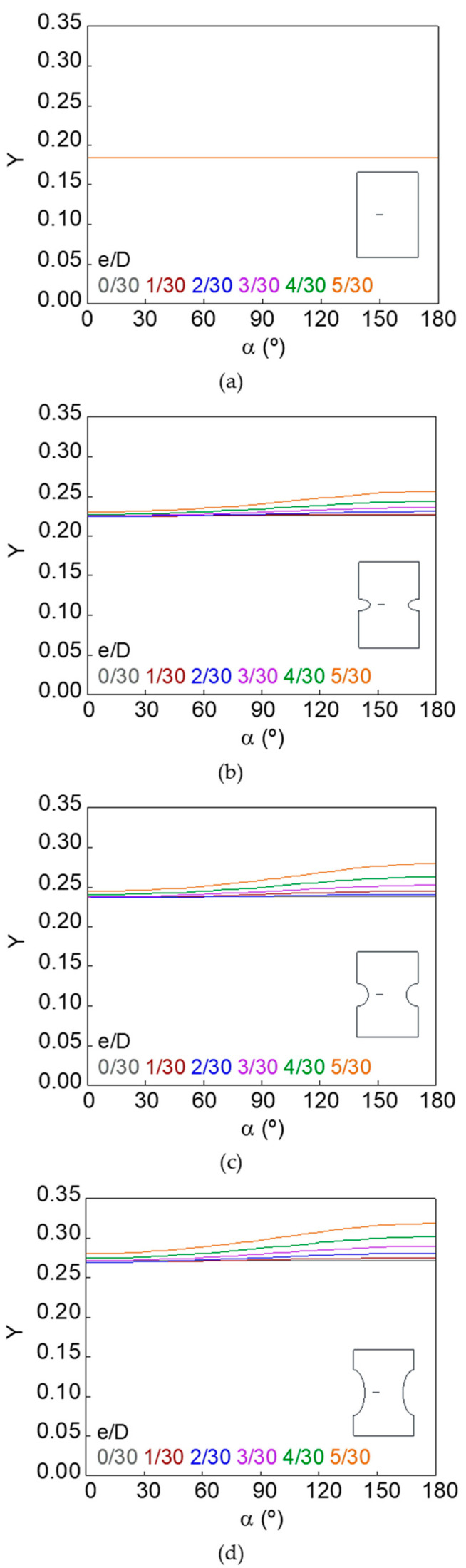
Dimensionless SIF along the crack front, *d*/*D* = 5/30: (**a**) smooth bar; (**b**) sharp notched bar; (**c**) circular notched bar; (**d**) blunt notched bar.

**Figure 9:**
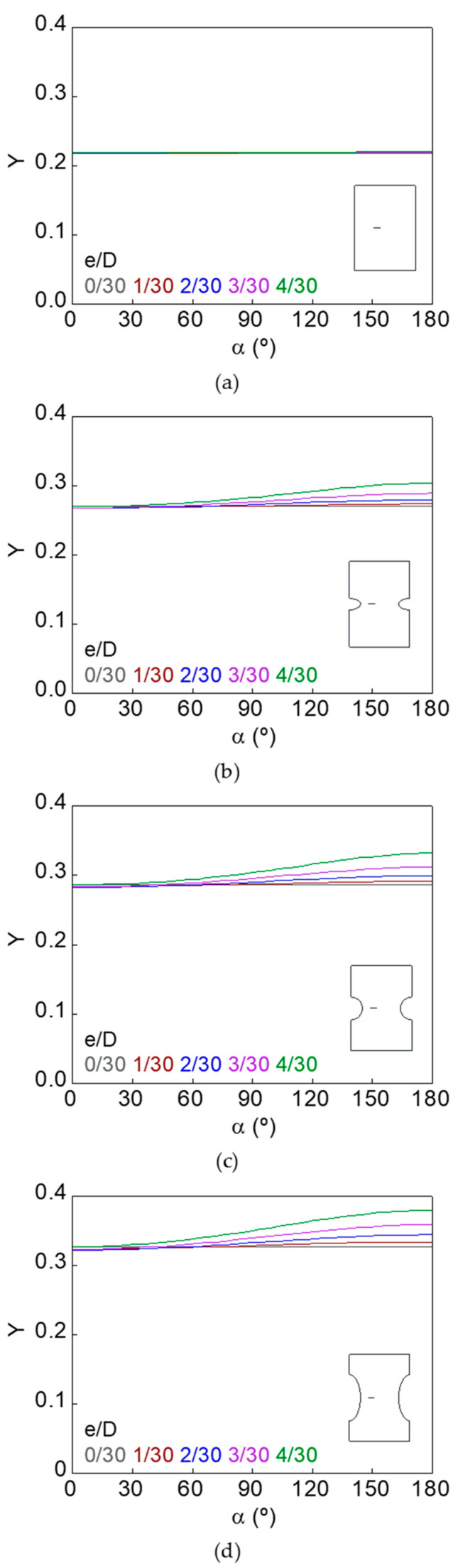
Dimensionless SIF along the crack front, *d*/*D* = 7/30: (**a**) smooth bar; (**b**) sharp notched bar; (**c**) circular notched bar; (**d**) blunt notched bar.

**Figure 10:**
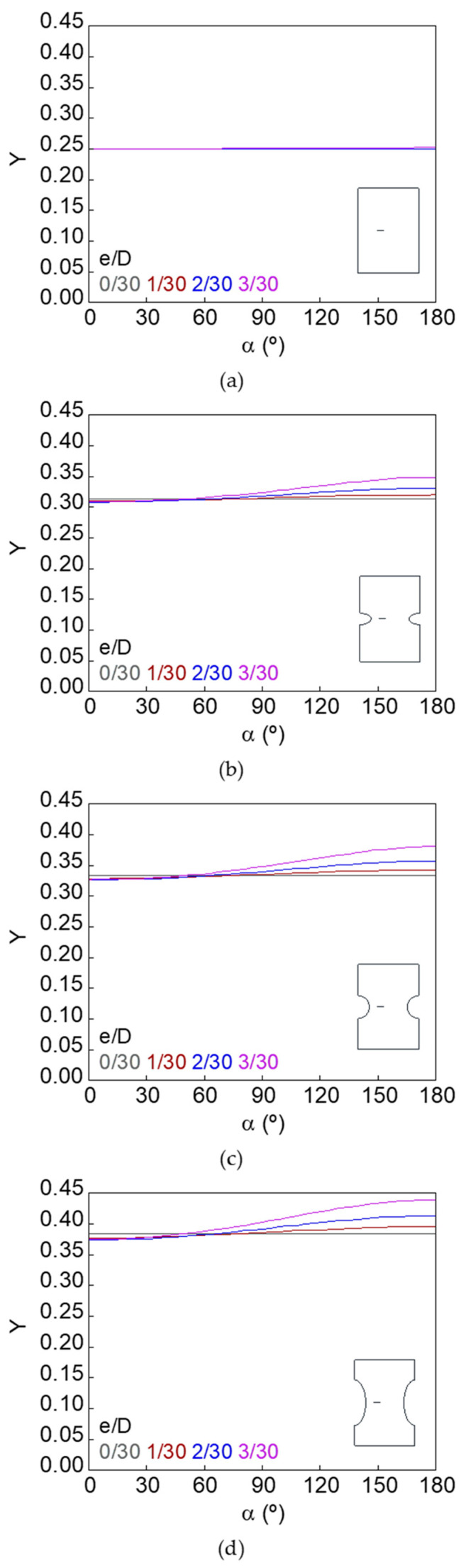
Dimensionless SIF along the crack front, *d*/*D* = 9/30: (**a**) smooth bar; (**b**) sharp notched bar; (**c**) circular notched bar; (**d**) blunt notched bar.

**Figure 11:**
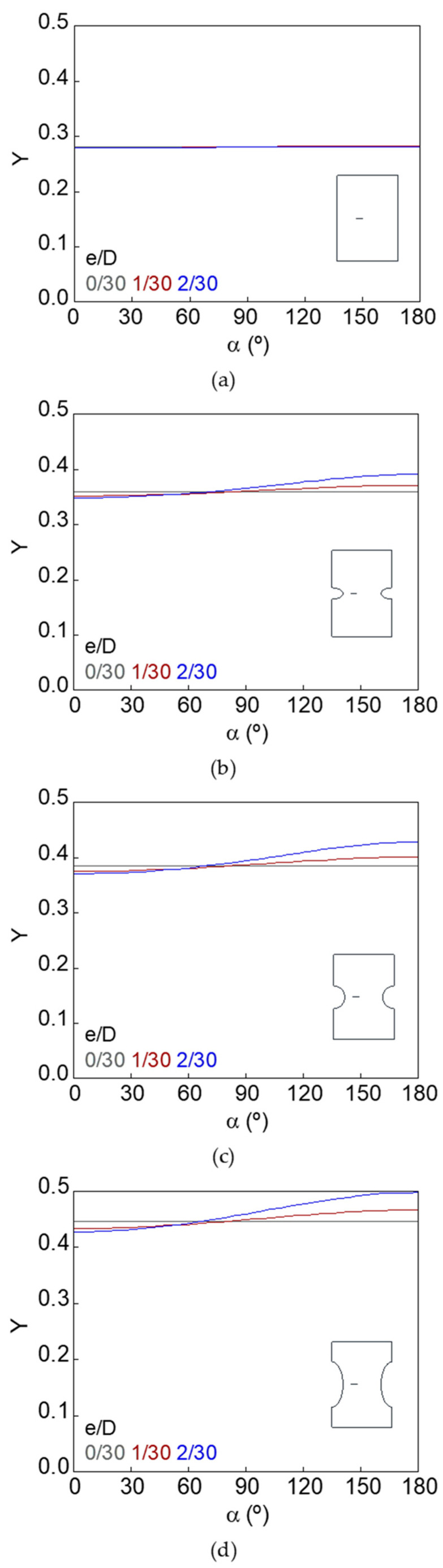
Dimensionless SIF along the crack front, *d*/*D* = 11/30: (**a**) smooth bar; (**b**) sharp notched bar; (**c**) circular notched bar; (**d**) blunt notched bar.

**Figure 12:**
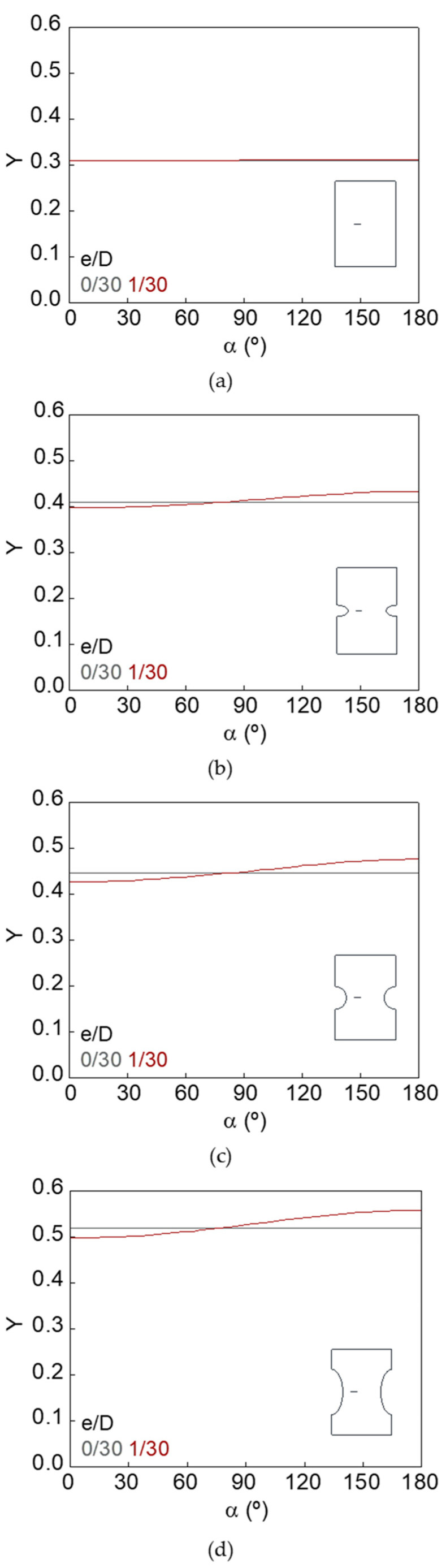
Dimensionless SIF along the crack front, *d*/*D* = 13/30: (**a**) smooth bar; (**b**) sharp notched bar; (**c**) circular notched bar; (**d**) blunt notched bar.

The dimensionless SIF along the crack front (*Y*–*α* curves) for smooth and notched bars with cracks of different geometries (defined by the relative crack diameter *d*/*D* and the relative crack eccentricity *e*/*D*) is shown in Figure 7, Figure 8, Figure 9, Figure 10, Figure 11 and Figure 12.

In the case of the smooth bar, the increase in eccentricity does not produce a variation in the SIF value along the crack front, because the crack is far enough away from the free surface of the bar not to be affected by it. On the other hand, for the notched specimens, a continuous increase in the SIF value is observed from the point farthest from the free surface of the bar (minimum SIF) to the point closest to it (maximum SIF). The maximum SIF and the difference between maximum and minimum SIF (caused by the crack eccentricity in relation to the bar axis) both increase with the relative crack diameter, with the relative crack eccentricity, and with the elliptical notch axial semi-axis *c*.

**Figure 13:**
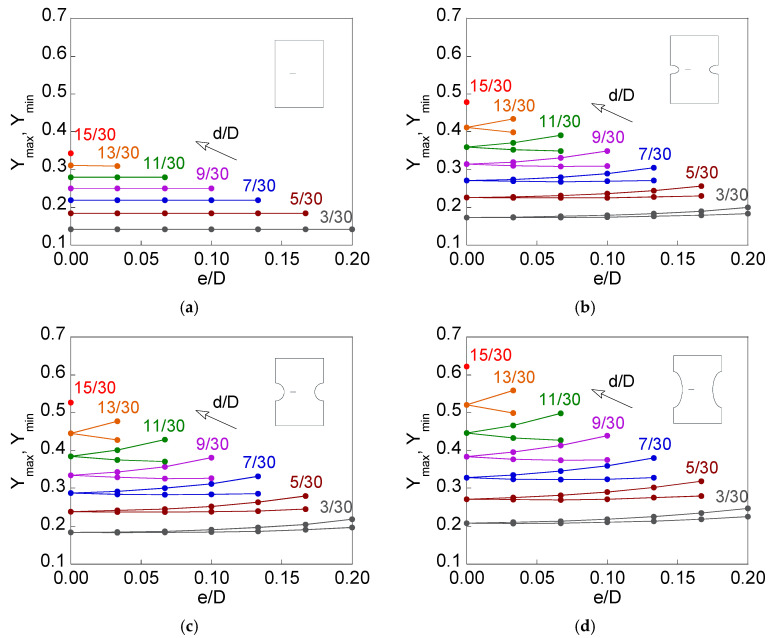
Maximum and minimum dimensionless FIT vs. relative crack eccentricity for several relative crack diameters: (**a**) smooth bar; (**b**) sharp notched bar; (**c**) circular notched bar; (**d**) blunt notched bar.

Figure 13 shows the maximum and minimum dimensionless SIFs (*Y*_max_ and *Y*_min_) as a function of relative crack eccentricity *e*/*D* for the relative crack diameters *d*/*D* studied and the four types of proposed specimens (smooth bar, sharp notched bar, circular notched bar, and blunt notched bar). The maximum SIF increases with the relative crack eccentricity, while the minimum SIF decreases for small relative crack eccentricities (more visible for high relative crack diameters) and increases for high relative crack eccentricities (only reached for small relative crack diameters).

The effect produced by the notches on the SIF is attributed to the loss of crack front constraint (due to the lack of material by the notch) and the appearance of a small bending in the specimen when the force is applied (due to the asymmetry generated by the eccentricity of the crack [14]). Both phenomena, constraint loss and bending, are more noticeable when the elliptical notch axial semi-axis is larger, if the elliptical notch radial semi-axis (notch depth) is kept constant. For small relative crack diameters *d*/*D*, the minimum SIF *Y*_min_ experiences an increase with the growth in crack eccentricity from a certain value of it, i.e., it presents a minimum (more noticeable for the blunt notched bar *c*/*b* = 2). This is due to the fact that, for a given value of relative crack diameter *d*/*D*, as the eccentricity increases the position of the point corresponding to the crack front farthest from the free surface of the bar, it begins to reduce its distance from the surface of the bar in the opposite position. Thus, the influence on the minimum SIF of the adjacent free surface is less, but from a certain eccentricity, the effect on the minimum SIF of the opposite bar surface begins to be noticed producing a growth in the minimum SIF.

### 3.3. Eccentric Circular Inner Crack Propagation

The propagation of circular inner cracks in notched bars under cyclic tensile loading has been modeled. It is considered that each point of the crack front propagates by fatigue perpendicularly to such a crack front, maintaining a circular geometry during its growth and following the law of Paris [25],

(5) $$\frac{da}{dN}=C\Delta K^{m}$$

where *C* and *m* are the Paris parameters characteristic of the material. For the modeling, the Paris exponent *m* = {2 and 3} has been chosen, among which the values corresponding to steels are usually found.

Only the points of the crack front of maximum and minimum value of the SIF (diametrically opposed) have been considered. The calculation has been performed incrementally, keeping constant the maximum crack advance Δ*a*_max_ (corresponding to the point closest to the free surface) and obtaining the minimum crack advance Δ*a*_min_ (corresponding to the point farthest to the free surface) based on the relationship between the SIFs according to Paris law [25]:

(6) $$\Delta a_{\min}=\Delta a_{\max}{\left(\frac{Y_{\min}}{Y_{\max}}\right)}^{m}$$

where the maximum and minimum dimensionless SIF, *Y*_max_ and *Y*_min_, have been obtained from fitting to third-degree polynomial equations (with the least squares method) of the dimensionless SIF results obtained as a function of the relative crack diameter (*d*/*D*) and the relative crack eccentricity (*e*/*D*).

**Figure 14:**
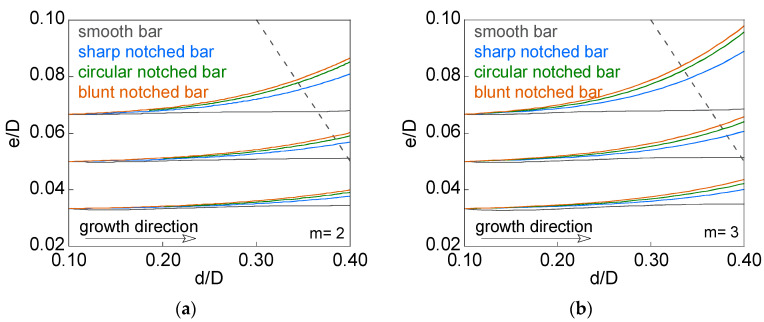
Fatigue propagation for three cracks with initial geometries (*d*/*D*)_0_ = 3/30 and (*e*/*D*)_0_ = {1/30, 1.5/30, and 2/30}: (**a**) *m* = 2; (**b**) *m* = 3.

For the four types of specimens (smooth bar and notched bar with *c*/*b* = {0.5, 1, and 2}), Figure 14 shows the curves’ relative crack eccentricity versus relative crack diameter (*e*/*D*-*d*/*D* curves) corresponding to the propagation of three cracks with initial geometries of equal diameter (*d*/*D*)_0_ = 3/30 and different eccentricity (*e*/*D*)_0_ = {1/30, 1.5/30, and 2/30} in materials with the Paris exponent *m* = {2 and 3}. The value of the maximum crack advance, kept constant throughout the whole propagation process and obtained through a convergence study, was Δ*a*_max_/*D* = 0.05/30. The limits associated with the configurations studied are represented in Figure 14 with a gray dashed line.

In the case of the smooth specimen, there is no change in the eccentricity of the crack during fatigue propagation, because the size of the defect is too far from the surface of the bar. On the other hand, in the notched specimens, a gradual increase in eccentricity is observed during fatigue propagation, this being greater as the initial relative crack eccentricity increases (for the same initial relative crack diameter). Regarding the geometry of the notch, the rise in the axial semi-axis *c* of the elliptical notch increases the eccentricity of the crack during its propagation due to the relationship between the maximum and minimum SIFs reached in each crack front. Finally, higher values of the Paris exponent *m* (characteristic of the material) produce higher relative crack eccentricity during fatigue propagation according to the Paris law.

## 4. Conclusions

The following conclusions have been obtained regarding notch effects on the stress intensity factor and on the fatigue crack path for eccentric circular internal cracks in elliptically notched round bars under tensile loading:

- The presence of the notch in the cracked bar produces an increase in the stress intensity factor (SIF) value.
- For elliptical notches of equal depth, the increase of the axial semi-axis raises the SIF for the same crack configuration.
- The cause of this phenomenon can be attributed to the constraint loss on the crack front. This fact also favours the specimen bending due to the crack eccentricity.
- The maximum SIF increases with the relative crack eccentricity, while the minimum SIF decreases for small eccentricities and increases for high eccentricities.
- The difference between the SIF values in the crack front increases with the relative crack diameter, the relative crack eccentricity, and the notch axial semi-axis.
- During fatigue propagation of small eccentric cracks, there is a gradual increase in eccentricity, this effect being more visible with increasing initial relative crack eccentricity, notch axial semi-axis, and Paris exponent.

## Figures and Tables

**Table 1:** Diameters and eccentricities of the circular inner crack.

*d* (mm)	*e* (mm)
0.3	0; 0.1; 0.2; 0.3; 0.4; 0.5; 0.6
0.5	0; 0.1; 0.2; 0.3; 0.4; 0.5
0.7	0; 0.1; 0.2; 0.3; 0.4
0.9	0; 0.1; 0.2; 0.3
1.1	0; 0.1; 0.2
1.3	0; 0.1
1.5	0

**Table 2:** Number of mesh elements.

Notch and Crack Geometry			Number of Elements		
*c/b*	*d/D*	*e/D*	Mesh A	Mesh B	Mesh C
1	3/30	6/30	4400	5840	8320
	13/30	1/30	5700	7240	10,120

## Data Availability

Not applicable.
